# Mothers' cord care practices in an academic hospital in Kenya

**DOI:** 10.4314/ahs.v23i1.45

**Published:** 2023-03

**Authors:** O'Brien M Kyololo, Maculater J Kipkoech

**Affiliations:** 1 School of Nursing & Midwifery, Moi University, Eldoret, Kenya; 2 Department of Health, Uasin Gishu County, Eldoret, Kenya

**Keywords:** Cord care, practices, neonates, mothers, Kenya

## Abstract

**Background:**

Nearly 99% of neonatal deaths globally occur in low- and middle-income countries with about three-quarters of the neonatal deaths resulting from sepsis including those arising from cord infections. Thus, good cord care practices have the potential to reduce the neonatal deaths in low and middle-income countries such as Kenya.

**Objective:**

Describe cord care practices of mothers in an academic hospital in Kenya.

**Methods:**

A questionnaire was administered to 114 mothers attending child welfare clinic at 6 weeks in an academic hospital in Western Kenya. Descriptive statistics were computed for continuous variables while frequencies were computed for categorical variables. Parametric and non-parametric tests were used to check for association between maternal variables and cord care practices.

**Results:**

Most mothers applied chlorhexidine (n =73, 64%) or practiced dry cord care (n = 17, 14.9%). Some mothers (12.9%) applied potentially harmful substances including saliva, ash and soil. Mothers who attended at least three antenatal clinic visits practiced the recommended cord care (χ^2^ =16.02, p. = 0.03).

**Conclusions:**

Although mothers predominantly practiced the recommended cord care, some potentially deleterious practices were reported. There is need to encourage attendance to antenatal clinic in order to optimize umbilical cord care practices.

## Introduction

Approximately 4 million neonatal deaths occur globally each year with about 99% of the deaths occurring in low and middle income countries (LMIC) particularly in sub-Saharan Africa (SSA).^1^,^2^ About three-quarters of these neonatal deaths occur as a result of sepsis^3^,^4^ with umbilical cord infections (omphalitis) accounting for a significant proportion of the infections in SSA.^5^–^7^ Empirical evidence suggests that these preventable neonatal deaths can be significantly reduced with improved care around the time of birth and by observing optimal umbilical cord care practices during the first week of life.^8^,^9^ To minimize the neonatal mortality associated with poor umbilical cord care practices, the World Health Organization (WHO) recommends dry umbilical cord care that entails keeping the cord clean without application of anything, exposing the cord to air or loosely covering it with a clean cloth, and only cleaning with sterile water when soiled.^10^ Use of topical antiseptics may, however, be used on the umbilical cord stump in settings with high infection rates and/or poor hygienic conditions.^10^,^11^ Despite these recommendations, diverse traditional cord care practices continue to be reported among mothers globally^12^–^14^ with some of the cord care practices such as the application of herbs, cow dung, ghee and saliva likely to cause infections.^8^,^9^ Sadly, these harmful cord care practices are largely reported in countries with some of the highest neonatal mortality rates globally.^15^,^16^

The neonatal mortality in Kenya is estimated at 22 for every 1000 live births,^17^ with about 20% of these deaths being attributed to infections including umbilical cord infections.^8^,^18^ Thus, good cord care practices are fundamental to achieving a reduction in the unacceptably high neonatal death rate in Kenya.^7^,^19^ In recognition of umbilical infections as a major contributor to the neonatal deaths in the country, practice guidelines on cord care have been developed.^20^ The overarching emphasis of the guidelines is the use of 4% chlorhexidine on the umbilical stump which should be initiated at the health facility and continued by the mother for seven days or until the cord stump falls off.

In view of the contribution of cord infections to the neonatal mortality in Kenya and the critical role of mothers in ensuring optimal cord care and, consequently, a reduction in neonatal infections, this study was conducted to determine the cord care practices of mothers in an academic hospital in Kenya.

## Methods

### Design and Setting

We conducted a descriptive survey between November 2018 and January 2019 in a large academic hospital in Western Kenya. The hospital has a detached mother and baby unit where mothers are attended to during pregnancy and birth. An average of 14, 000 deliveries are conducted in the unit annually. It is in the same mother and baby unit where postnatal care and vaccination services for infants are provided.

### Participants

Purposive convenience sampling was used to recruit postnatal mothers who attended child welfare clinic (CWC) at 6 weeks after birth. In Kenya, it is a government policy that mothers bring their babies to the CWC at the age of 6 weeks for growth monitoring and vaccination. It is this maternal population that was the target of our study. To identify potential study participants, the CWC register and clinic cards were scrutinized to identify babies being brought for follow up at the age of six weeks. Mothers attending the CWC during the study period, irrespective of whether they delivered in the hospital or not, were recruited. We excluded mothers who were sick and/or whose baby was sick and needed medical treatment.

### Procedures

A questionnaire that was previously used in a similar study^21^ was adopted and modified for data collection. Additional items on who initiated cord care and whether information on cord care was given after the birth were included in the questionnaire. The final study tool was pilot-tested for reliability with a Cronbach's alpha of 0.76. The second author (MK) administered the questionnaires. The CWC register and clinic cards were used to identify mothers who met the inclusion criteria. Those who met the criteria were given detailed information about the study. Mothers who verbalized willingness to participate in the study were requested to sign a consent form before being interviewed in an isolated corner in the waiting bay.

### Analysis

Data were analysed descriptively into mean, standard deviation, median and interquartile range for continuous data. Chi-square test was used to check for association between categorical data and cord care. Independent sample t-test was used to compare means for continuous data while Fisher's exact test was used to identify factors associated with cord care. P-value < 0.05 was specified as statistically significant.

## Results

The 114 mothers who completed the survey were aged 18 - 41 years (M = 27±4.7 years). The median number of children the respondents had was 1 (IQR 0, 2; range: 0-4). On average, the mothers had made an average of 4.5 (SD = 0.9, range: 3-7) antenatal clinic visits during the pregnancy with most of the mothers making the first antenatal clinic visit during the 2nd trimester (52.6%, n = 60). All the mothers had delivered in a health facility; either in the study hospital (n = 102; 89.5%) or in a community health centre (Table 1).

**Table 1:** Demographic Characteristics of Sample

	n	(%)	Mean	SD	Median	IQR
**Age (yrs.)**			27	4.7		
**No. of children**			0.98	1.07	1	0, 2
**ANC visits**			4.5	0.9		
**Marital status**						
Married	106	93.0				
Single	8	7				
**Level of Education**						
Primary	22	19.3				
Secondary	28	24.6				
Tertiary	64	56.1				
**Occupation**						
Employed	42	36.8				
Unemployed	44	38.6				
Housewife	28	24.6				
**1^st^ ANC visit timing**						
1^st^ trimester	54	47.3				
2^nd^ trimester	60	52.6				
**Birth attendant**						
Nurse	64	56.1				
Doctor	50	43.9				

**Abbreviations:** SD; Standard Deviation; IQR: Interquartile Range

### Cord care practices

Most mothers used either chlorhexidine (n = 73; 64%) or surgical spirit (n = 9; 8%) on the cord with most mothers preferring to apply the antiseptic agent twice daily. Four fifths of the mothers (n = 91) reported that they applied the diaper below the umbilicus and a similar proportion (78%; n = 89) wiped the baby during bath (Table 2).

**Table 2:** Cord Care Practices

	n	%
**Taking care of the cord**		
Uncover	83	73
Cover	20	18
Clean with surgical spirit	10	9
**Substance applied on cord stump**		
Chlorhexidine	73	64
Surgical spirit	10	9
Saliva	6	5.2
Breast milk	4	3.5
Others*	4	3.5
No substance applied	17	14.9
**Frequency of application of substance**		
1	17	17.5
2	64	66.0
3	16	16.5
**Application of diaper**		
Below umbilicus	91	79.8
Above umbilicus	23	20.2
**Initiator of cord care**		
Self	69	60.5
Nurse	14	12.3
Others	14	12.3
Doctor	2	1.8
No response	15	13.2
**Care of the cord during bath**		
Wiped	89	78
Immersed in water	25	22

* Includes 2 for ash and one each for soil and shea butter

### Factors associated with cord care practices

The number of antenatal clinic visits were associated with cord care practices. Mothers who attended at least four ANC visits were more likely to practice the recommended cord care (χ 2 =16.02, p. = 0.03) (Table 3).

**Table 3:** Factors Associated with Cord Care Practices

	Appropriate cord care		*p*-value
	No	Yes	
**Age (yrs.)**	27.5 (0.92)	26.1 (0.49)	0.14†
**Marital status**			0.49§
Married	28 (26.4)	78 (73.4)	
Single	3 (37.5)	5 (62.5)	
**Occupation**			0.89‡
Employed	11 (26.2)	31 (73.8)	
Unemployed	13 (29.5)	31 (70.5)	
Housewife	7 (25)	21 (75)	
**Number of children**			0.234§
0	11 (22.9)	37 (77.1)	
1	7 (19.4)	29 (80.6)	
2	7 (43.8)	9 (56.3)	
3	5 (41.7)	7 (58.3)	
4	1 (50)	1 (50)	
**ANC visits**			0.03§
3	7 (58.3)	5 (42.7)	
4	12 (23.1)	40 (76.9)	
5	4 (13.5)	28 (87.5)	
6	6 (37.5)	10 (62.5)	
7	2 (100)	0 (0)	
**Timing of 1^st^ ANC visit**			0.89‡
1^st^ trimester	15 (27.8)	39 (72.2)	
2^nd^ trimester	16 (26.7)	44 (73.3)	
**Birth Assistant**			0.86‡
Nurse	17 (26.6)	47 (73.4)	
Doctor	14 (28)	36 (72)	

† t-test

§ Fishers' exact test

‡ Chi-square

## Discussion

Infections including omphalitis continue to be a main cause of neonatal mortality globally and more so in resource-limited countries where the incidence is dishearteningly high.^5^,^22^ With optimal cord care practices these infections can, however, be significantly reduced. It is in recognition of the critical role of umbilical cord care that national^20^,^23^ and international organizations^22^,^24^ have developed divergent recommendations for cord care. The overarching emphasis of these recommendations is dry cord care for babies born in a hospital or in settings with low neonatal mortality and application of antiseptic solutions (mainly chlorhexidine solution or gel) for babies born at home or in settings with high neonatal mortality rates.^22^,^23^

Mothers in our study mainly used chlorhexidine solution or surgical spirit for cord cleaning (73%) which mirrors what has been reported in other parts of the country^19^ as well as in Uganda,^25^ Tanzania,^26^ Ghana,^27^ Nigeria,^11^,^28^ Benin^29^ and Nepal.^30^ We also noted a significant proportion of mothers who reported to have practiced dry cord care (15%) which is consistent with the finding in earlier studies in the country.^7^,^19^ Studies in other low and middle-income countries have also reported dry cord care practice among mothers.^11^,^31^ For instance, 8% of Ghanaian mothers^27^ and 27% of rural Indian mothers kept the cord stump dry without application of any substance.^32^ Although it is not clear why such a high proportion of the Indian mothers appeared to observe the recommended practice compared to other LMICs, it is noteworthy that mothers who were assisted by a skilled attendant at birth (70%) were two times more likely to practice dry cord care.^32^ Similar to the Indian study, all mothers in our study reported to have delivered in a health facility which could explain the high number of mothers who practiced the recommended cord care – use of chlorhexidine or dry cord care.^10^,^22^ It is at the hospital where health care providers are expected to initiate appropriate cord care and ensure that mothers continue with the same care after discharge.^24^ Similarly, the reported use of breast milk on the cord stump, albeit by a small proportion of mothers, is commendable considering the evidence that its application has the potential to reduce cord separation time and to minimize bacterial colonization on the cord.^34^

Similar to earlier studies in the country^7^, ^19^ we observed a disheartening trend whereby mothers used potentially harmful substances including, saliva, ash and dust on the cord stump. The use of these and other potentially harmful substances is not limited to the country.^15^,^33^ Application of powder and lizard droppings on the cord has been a common practice in Uganda,^35^ herbs, chicken faeces, brick ash and python oil have been used in Zambia,^36^ petroleum jelly, butter and hair lotion have been used in Ethiopia^31^,^37^ while the use of mustard oil, herbs and chewed rice have been used variably in Nepal and Bangladesh.^8^,^38^ Although the harmful effects of these substances has not been comprehensively examined, 8,37 the risk for infections they pose to the neonates is well documented.^39^ Empirical evidence shows that the application of unhygienic substances such as soil, saliva and cow dung on the umbilical cord stump poses the risk for tetanus on the neonates.^40^–^43^

Four fifths of mothers in our study applied the diaper below the umbilicus and practiced sponge bath until the cord detached reportedly to facilitate healing and prevent contamination of the umbilical stump. These are encouraging statistics considering that they are consistent with the recommendations of numerous cord care guidelines.^10^,^23^ Other studies in peri-urban settings in the country have, however, reported lower frequency of these practices with 55-65% of mothers sponge-bathing and applying the diaper below the umbilicus.^19^ Mothers in other LMICs including in Nigeria,^11^,^44^ Pakistan^45^ and Ghana^14^ have also reported sponge-bathing and keeping the cord uncovered albeit at a lower frequency than reported in our study. The fact that all mothers in our study had delivered in hospital could explain the higher frequency of their practice with respect to sponge bathing and application of the diaper since it is expected that health care providers would introduce, and demonstrate to, mothers to all aspects of recommended cord care following after birth.^10^

Our findings showed that the number of antenatal clinic visits were associated with cord care practices; mothers who attended four or more ANC visits were more likely to practice the recommended cord care. This positive relationship between antenatal follow-up and good cord care is not unique to the Kenyan setting.^46^–^48^ For instance, Ghanaian mothers who received adequate antenatal care were 4 times more likely to practice the recommended cord care.^14^ Although we did not explore why more antenatal care visits would result in better cord care practices, it is expected that the more expectant women interact with health care providers during the prenatal period the more they are provided with information and made aware of the recommended cord care.

Although our study has shed light on a critical component of newborn care in a region that has not been comprehensively studied, our findings may be limited from several fronts. The sample size was relatively small and the sampling technique may have left out mothers with divergent views from the recruited sample thus limit the generalizability of the result. Additionally, due to the study design, it is impossible to draw any causal associations from our findings. Furthermore, the use of a researcher-administered questionnaire poses the risk of social desirability bias.

## Conclusion

Although, generally, mothers practiced the recommended cord care, instances of potentially harmful cord care practices were noted. Increasing the number of antenatal clinic visits has the potential to improve cord care practices and overall outcomes of neonates. The need for a comprehensive understanding of the cord care practices of mothers warrant large-scale multi-site observational studies in the country.
